# The hemoglobin-red blood cell distribution width ratio is a reliable predictor of recurrence of ketoacidosis in patients with type 2 diabetes

**DOI:** 10.3389/fendo.2026.1772128

**Published:** 2026-03-24

**Authors:** Chenhong Zhang, Hao Huang, Xiaosheng Zhu, Weiwei Tang, Lei Cao, Chun Chen, Zhaoqing Bai

**Affiliations:** Emergency Department, Anqing Municipal Hospital, Anqing, Anhui, China

**Keywords:** biomarkers, complications, diabetes, HbA1c, inflammation, metabolism, recurrence rate, red blood cells

## Abstract

**Background:**

Diabetic ketoacidosis is a common disease in the emergency department, characterized by severe metabolic disorders, which poses a significant threat to the life and health of patients with type 2 diabetes. Hemoglobin reflects the body’s oxygen-carrying capacity and nutritional status, while the red cell distribution width indicates the heterogeneity in the size of red blood cells. The ratio of these two indicators can comprehensively reflect the systemic metabolic status and inflammatory state of patients with diabetic ketoacidosis.

**Objectives:**

To study the association between the HRR and the recurrence of diabetic ketoacidosis.

**Methods:**

A retrospective study was conducted on patients with type 2 diabetic ketoacidosis at a local medical center. The HRR ratio at the time of the patients’ first admission was collected. Statistical analysis was used to examine the relationship between the HRR ratio and the recurrence of DKA.

**Results:**

Logistic regression analysis revealed that HRR was associated with the recurrence of DKA. As the HRR value increased, the recurrence rate of DKA decreased. Additionally, ROC curve analysis indicated that the HRR ratio had good sensitivity and specificity in predicting the recurrence of type 2 diabetic ketoacidosis, with sensitivity values of 76.00% and 74.70%, respectively.

**Conclusion:**

HRR is a reliable predictor for the recurrence of DKA.

## Introduction

Diabetes mellitus (DM) is a life-threatening chronic disease, with type 2 diabetes being the most prevalent subtype that imposes a substantial burden on global healthcare systems. Given the future trends of population growth, aging and urbanization, by 2050, the number of global diabetes patients is expected to increase by 45%, reaching 853 million people (1). Without effective management, diabetic patients are susceptible to various complications, among which diabetic ketoacidosis (DKA) is a common and acute life-threatening condition. Clinically, DKA is characterized by hyperglycemia (serum glucose > 250 mg/dL), increased anion gap metabolic acidosis (anion gap > 10-12, serum bicarbonate < 18 mEq/L and/or pH < 7.3), and ketosis (ketonuria and/or ketonemia) (2, 3). The incidence of diabetic ketoacidosis (DKA) in patients with T2DM is relatively high, which also imposes a considerable clinical burden (4, 5). In the United States alone, there are over 140,000 DKA-related hospitalizations annually, resulting in a total annual healthcare cost of 240 million US dollars. Notably, the treatment costs for patients with repeated DKA episodes account for a significant proportion of this economic burden (6). While hospital readmissions due to DKA contribute substantially to the healthcare burden, a considerable number of these repeated episodes are potentially preventable. At present, reliable predictive markers for DKA recurrence are scarce. Therefore, identifying a reliable, cost-effective, and easily accessible biomarker for the early assessment of DKA relapse risk remains a critical challenge in clinical practice. Addressing this gap is of paramount importance for reducing DKA relapse rates and improving patient prognosis.

Hemoglobin (HGB) is a specialized protein in red blood cells responsible for oxygen transport, which imparts the red color of blood and consists of globin and heme moieties. Red blood cell distribution width (RDW) is a hematological parameter reflecting the heterogeneity of red blood cell sizes, typically quantified as the coefficient of variation of measured red blood cell dimensions. In recent years, the hemoglobin-to-red blood cell distribution width ratio (HRR) has emerged as a promising marker that integrates information on overall red blood cell health and functional status. Notably, HRR offers the advantages of being economical, cost-effective, and readily accessible in clinical settings, and has been investigated as a potential biomarker for various diseases, including myocarditis and peripheral artery disease (7–9). As a globally prevalent disorder, DM is also frequently associated with concurrent red blood cell dysfunction (10). However, existing red blood cell-related biomarkers lack sufficient capacity to explain the risk of DKA relapse and associated patient prognosis. Against this background, we propose HRR as a novel inflammatory biomarker, with the aim of evaluating its correlation with DKA relapse in patients with type 2 diabetes mellitus (T2DM).

## Research methods

### Research subjects

We retrospectively collected data from patients with T2DM complicated by DKA who were admitted to local medical center for the first time over the past several years. The diagnoses of T2DM and DKA were made in accordance with the diagnostic criteria published by the American Diabetes Association in Diabetes Care in 2025 (11, 12). Patients meeting any of the following criteria were excluded from the study: type 1 diabetes mellitus, gestational diabetes mellitus, specific types of diabetes, severe liver disease, history of gastrointestinal surgery, severe mental disorders, immunodeficiency, malignant tumors, coronary heart disease, stroke, history of cardiopulmonary resuscitation, exocrine pancreatic diseases, secondary diabetes induced by drugs or endocrine disorders, patients who denied the first episode of DKA, incomplete clinical data, and inability to cooperate with the completion of the study. All study procedures were performed in compliance with relevant guidelines and regulations, including the Declaration of Helsinki. Informed consent was obtained from all participants prior to their inclusion in the study.

### Research methods

This study was a retrospective investigation conducted on patients with T2DM complicated by DKA who were admitted to our hospital over the past several years. We collected the following clinical and laboratory data from eligible patients: hematological parameters [hemoglobin (HGB), red blood cell distribution width (RDW), white blood cells (WBC), lymphocytes (LYM), neutrophil percentage (NEU%)], inflammatory marker [C-reactive protein (CRP)], metabolic indicators [ketone bodies, cholesterol (CHOL), triglycerides (TG), lactic acid (Lac), glycated hemoglobin A1c (HbA1c), fasting plasma glucose (FPG)], liver and kidney function parameters [alanine aminotransferase (ALT), aspartate aminotransferase (AST), serum creatinine (Scr), lactate dehydrogenase (LDH)], treatment-related information (insulin use), lifestyle factors (dietary control, smoking and drinking history), anthropometric index [body mass index (BMI)], and comorbidities. The HRR was calculated using the measured HGB and RDW values. For patients with multiple hospitalizations due to DKA, only data from their first admission were included in the analysis. All laboratory variables were collected within 24 hours of the patient’s hospitalization, and all aforementioned information was retrieved from the hospital’s electronic medical record system.

**Figure 1:**
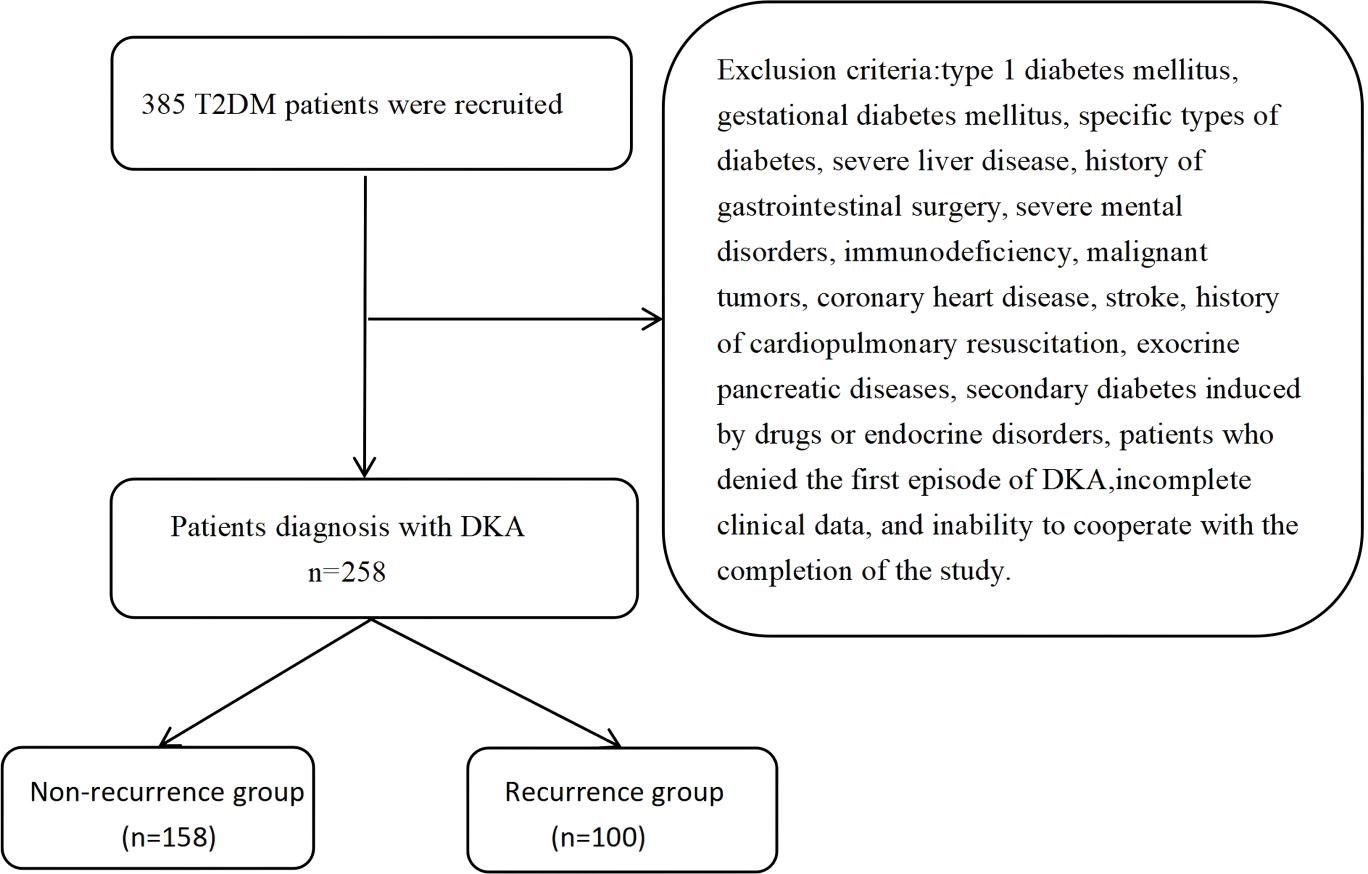
Flowchart for patient selection.

Patients were divided into two groups based on the recurrence of DKA: the recurrence group and the non-recurrence group. The primary outcome measure was DKA recurrence. Statistical analysis was performed to explore the associations between HRR, DKA recurrence, and long-term prognosis. The flow chart of data extraction was depicted in **Figure 1**.

### Statistical analysis

All statistical analyses were performed using SPSS 27.0 software. Measurement data conforming to a normal distribution were expressed as mean ± standard deviation ( X ± S), with independent samples t-tests used for comparisons between two groups and analysis of variance for comparisons among multiple groups. Measurement data not meeting the normal distribution were presented as quartiles [P50 (P25, P75)], and comparisons were conducted using the Mann-Whitney U test or Kruskal-Wallis H test. Categorical data were expressed as counts and percentages (n, %), and analyzed using the chi-square test. Stratified logistic regression analysis was employed to identify risk factors for DKA recurrence. Receiver operating characteristic (ROC) curves were constructed to evaluate the predictive value of HRR for DKA recurrence. A two-tailed P value < 0.05 was considered statistically significant.

## Results

### Comparison of general characteristics between the recurrence group and the non-recurrence group

A total of 258 patients with T2DM were enrolled in this study, including 144 males (55.81%) and 114 females (44.19%). Based on DKA recurrence status, patients were divided into the recurrence group (n = 100) and the non-recurrence group (n = 158), with a mean age of 55.47 years across all participants.

Statistical analysis revealed that compared with the non-recurrence group, the recurrence group had significantly lower levels of HGB, RDW, FPG, proportion of patients with dietary control, and HRR, with all differences reaching statistical significance (P < 0.05). In contrast, the recurrence group exhibited significantly higher levels of CHOL, TG,HbA1c, LDH, as well as a higher proportion of patients with smoking and drinking habits, and these differences were also statistically significant (P < 0.05).

No significant differences were observed between the two groups in terms of WBC, NEU%, LYM, Lac, Scr, ALT, AST, BMI, insulin use, or the prevalence of comorbidities including COPD, hepatic insufficiency, CHF, ASCVD, and CKD (P > 0.05). Detailed results are presented in **Table 1**.

**Table 1:** The general characteristics of patients in the non-recurrence and recurrence groups.

Variables		Non-recurrence group (n=158),n(%)	Recurrence group (n=100),n(%)	t/z/χ²	P
Gender	Male	90(56.96)	54(54.00)	0.218	0.641
	Female	68(43.04)	46(46.00)		
Age (years)		56.37 ± 16.95	54.04 ± 18.02	1.051	0.294
HGB(g/L)		135.50(122.75, 156.00)	124.00(108.25, 141.00)	-4.556	0.000
RDW		45.20(42.50, 48.50)	43.45(39.95, 46.68)	-3.136	0.002
HRR		3.24(2.79,3.68)	2.63(2.09,3.12)	-7.284	0.012
WBC(×10^9^/L)		11.50(6.85, 16.25)	11.11(7.76, 15.17)	-0.217	0.828
LYM(×10^9^/L)		1.00(0.70, 1.53)	1.10(0.69, 1.70)	-1.193	0.233
NEU(%)		85.00(75.38, 89.33)	83.75(75.80, 88.60)	-0.291	0.771
CRP(mg/L)		22.61(3.83,102.55)	12.92(3.89,49.37)	-1.873	0.061
Ketone bodies(mmol/L)		4.05(1.52, 6.89)	4.39(2.01, 7.25)	-0.707	0.479
CHOL(mmol/L)		3.89(2.96, 4.90)	4.86(3.86, 6.36)	-5.678	0.000
TG(mmol/L)		1.39(1.02, 2.31)	2.70(1.78, 3.66)	-4.875	0.000
Lac(mmol/L)		2.07(1.56, 3.21)	2.67(1.53, 3.73)	-1.171	0.241
HbA1c(%)		10.50(8.90, 12.45)	12.20(10.36, 13.85)	-4.205	0.000
Scr(umol/L)		89.50(65.00, 128.50)	95.50(66.00, 144.25)	-0.636	0.525
ALT(IU/L)		17.00(11.00, 32.25)	14.50(10.00, 26.75)	-1.408	0.159
AST(IU/L)		21.50(15.00, 39.25)	19.00(13.00, 34.00)	-1.637	0.102
FPG(mmol/L)		17.44(12.16, 24.44)	13.77(11.73, 21.58)	-2.555	0.011
LDH(IU/L)		166.50(141.75, 266.25)	201.00(187.25, 238.75)	-3.019	0.003
BMI(Kg/m²)		23.25(20.30, 25.65)	24.05(20.31, 28.58)	-1.390	0.165
Insulin usage	No	94(59.49)	63(63.00)	0.316	0.574
	Yes	64(40.51)	37(37.00)		
Dietary control	Yes	125(79.11)	64(64.00)	7.140	0.008
	No	33(20.89)	36(36.00)		
Smoking and Drinking	No	129(81.65)	70(70.00)	4.709	0.030
	Yes	29(18.35)	30(30.00)		
Comorbidities:					
COPD	No	104(65.82)	69(69.00)	0.280	0.597
	Yes	54(34.18)	31(31.00)		
Hepatic insufficiency	No	156(98.73)	96(96.00)	2.015	0.156
	Yes	2(1.27)	4(4.00)		
CHF	No	146(92.41)	89(89.00)	0.874	0.350
	Yes	12(7.59)	11(11.00)		
ASCVD	No	151(95.57)	97(97.00)	0.336	0.562
	Yes	7(4.43)	3(3.00)		
CKD	No	151(95.57)	90(90.00)	3.086	0.079
	Yes	7(4.43)	10(10.00)		

HGB, hemoglobin; RDW, red blood cell distribution width; WBC, white blood cells; LYM, lymphocytes; NEU, neutrophil percentage; CRP, C-reactive protein; CHOL, cholesterol; TG, triglycerides; Lac, lactic acid; HbA1c, glycated hemoglobin A1c; Scr, serum creatinine; ALT, alanine aminotransferase; AST, aspartate aminotransferase; FPG, fasting plasma glucose; LDH, lactate dehydrogenase; BMI, body mass index; COPD, Chronic Obstructive Pulmonary Disease; CHF, congestive heart failure; ASCVD, Atherosclerotic cardiovascular disease; CKD, Chronic kidney disease.

### Analysis of recurrence rates and clinical characteristics at different levels of HRR

HRR was divided into four groups by quartiles: the first group (Q1) with 1.15 < HRR ≤ 2.54, the second group (Q2) with 2.54 < HRR ≤ 2.99, the third group (Q3) with 2.99 < HRR ≤ 3.44, and the fourth group (Q4) with HRR > 3.44. The DKA recurrence rates in the four HRR quartile groups were 54.69%, 41.94%, 38.24%, and 20.31%, respectively. A significant downward trend in DKA recurrence rate was observed with increasing HRR levels, and this trend was statistically significant (P < 0.05).

Concomitantly, as HRR levels increased across the quartile groups, the levels of WBC, CHOL, TG, HbA1c, and LDH gradually decreased. The differences in these indicators among the four HRR subgroups were statistically significant (P < 0.05). Detailed data are presented in **Table 2**.

**Table 2:** The recurrence rates and clinical characteristics of DKA in different HRR groups.

Variables		Q1(n=64)n(%)	Q2(n=62)n(%)	Q3(n=68)n(%)	Q4(n=64)n(%)	t/z/χ²	P
HRR range		(1.15,2.54)	(2.54,2.99)	(2.99,3.44)	(3.44,7.44)		
Gender	Male	37(57.81)	30(48.39)	40(58.82)	37(57.81)	1.844	0.605
	Female	27(42.19)	32(51.61)	28(41.18)	27(42.19)		
Age (years)		55.78 ± 19.54	58.50 ± 14.87	54.71 ± 16.45	53.03 ± 18.23	1.101	0.349
HGB(g/L)		125.50(106.00, 151.75)	128.00(121.00, 135.00)	135.00(125.00, 143.00)	149.50(109.25, 167.00)	5.896	0.117
RDW		43.75(40.43, 47.70)	45.25(43.28, 47.30)	43.50(41.33, 45.80)	45.30(41.30, 51.88)	5.889	0.117
WBC(×10^9^/L)		13.59(10.91, 16.97)	13.14(6.67, 16.68)	10.23(7.09, 15.43)	8.41(6.02, 12.71)	22.778	0.000
LYM(×10^9^/L)		0.90(0.62, 1.60)	1.00(0.60, 1.63)	1.10(0.73, 1.70)	1.10(0.80, 1.60)	2.264	0.520
NEU(%)		83.65(73.08, 88.83)	85.65(76.15, 88.85)	83.55(72.43, 90.08)	85.20(76.55, 89.08)	0.503	0.918
CRP(mg/L)		18.53(4.26,84.11)	20.94(4.02,86.88)	20.91(3.89,91.03)	12.08(2.87,55.44)	1.385	0.709
Ketone bodies(mmol/L)		2.61(1.39, 5.99)	5.01(1.57, 7.57)	5.20(2.66, 7.12)	4.28(1.71, 6.80)	7.102	0.069
CHOL(mmol/L)		5.07(4.14, 6.03)	4.41(3.83, 6.01)	3.59(2.85, 4.71)	3.74(2.80, 4.66)	48.760	0.000
TG(mmol/L)		2.69(1.84, 3.78)	2.27(1.44, 5.04)	1.96(1.34, 3.60)	0.98(0.76, 1.34)	70.132	0.000
Lac(mmol/L)		2.68(1.59, 3.67)	2.23(1.52, 3.63)	1.91(1.62, 3.37)	2.17(1.46, 3.44)	2.053	0.561
HbA1c(%)		12.32(10.58, 13.65)	11.40(9.33, 13.10)	10.90(9.23, 13.38)	10.32(8.85, 11.80)	15.392	0.002
Scr(umol/L)		106.50(70.00, 161.50)	99.50(65.75, 161.00)	82.50(64.00, 116.25)	91.00(63.25, 125.75)	5.807	0.121
ALT(IU/L)		17.00(10.00, 35.75)	16.00(10.00, 24.50)	14.50(12.00, 25.75)	16.50(11.00, 31.50)	0.467	0.926
AST(IU/L)		21.50(14.25, 45.00)	20.50(14.75, 39.00)	20.00(15.00, 35.50)	20.50(13.00, 38.75)	0.599	0.897
FPG(mmol/L)		14.76(10.70, 21.58)	15.52(12.27, 22.17)	14.57(12.04, 23.06)	17.62(14.59, 26.60)	6.632	0.085
LDH(IU/L)		236.50(201.25, 301.75)	204.00(157.50, 277.50)	188.50(158.25, 231.50)	168.50(141.00, 214.25)	43.992	0.010
BMI(Kg/m²)		24.85(21.43, 28.55)	22.55(19.78, 25.23)	22.90(20.30, 25.78)	23.80(20.38, 26.70)	6.826	0.078
Insulin usage	No	38(59.38)	37(59.68)	40(58.82)	42(65.63)	0.824	0.844
	Yes	26(40.63)	25(40.32)	28(41.18)	22(34.38)		
Dietary control	Yes	45(70.31)	43(69.35)	52(76.47)	49(76.56)	1.480	0.687
	No	19(29.69)	19(30.65)	16(23.53)	15(23.44)		
Smoking and Drinking	No	53(82.81)	50(80.65)	49(72.06)	47(73.44)	3.092	0.378
	Yes	11(17.19)	12(19.35)	19(27.94)	17(26.56)		
Comorbidities:							
COPD	No	44(68.75)	39(62.90)	46(67.65)	44(68.75)	0.661	0.882
	Yes	20(31.25)	23(37.10)	22(32.35)	20(31.25)		
Hepatic insufficiency	No	62(96.88)	59(95.16)	67(98.53)	64(100.00)	3.647	0.302
	Yes	2(3.13)	3(4.84)	1(1.47)	0(0.00)		
CHF(%)	No	57(89.06)	57(91.94)	61(89.71)	60(93.75)	1.097	0.778
	Yes	7(10.94)	5(8.06)	7(10.29)	4(6.25)		
ASCVD(%)	No	62(96.88)	58(93.55)	65(95.59)	63(98.44)	2.173	0.537
	Yes	2(3.13)	4(6.45)	3(4.41)	1(1.56)		
CKD(%)	No	60(93.75)	56(90.32)	65(95.59)	60(93.75)	1.508	0.680
	Yes	4(6.25)	6(9.68)	3(4.41)	4(6.25)		
Recurrence	No	29(45.31)	36(58.06)	42(61.76)	51(79.69)	16.287	0.001
	Yes	35(54.69)	26(41.94)	26(38.24)	13(20.31)		

HGB, hemoglobin; RDW, red blood cell distribution width; WBC, white blood cells; LYM, lymphocytes; NEU, neutrophil percentage; CRP:C-reactive protein; CHOL, cholesterol; TG, triglycerides; Lac, lactic acid; HbA1c, glycated hemoglobin A1c; Scr, serum creatinine; ALT, alanine aminotransferase; AST, aspartate aminotransferase; FPG, fasting plasma glucose; LDH, lactate dehydrogenase; BMI, body mass index; COPD, Chronic Obstructive Pulmonary Disease; CHF, congestive heart failure; ASCVD, Atherosclerotic cardiovascular disease; CKD, Chronic kidney disease.

### The relationship between HRR and the recurrence of DKA

Stratified logistic regression analysis was performed to investigate the association between HRR and DKA recurrence risk, with robustness of the association evaluated by stepwise incorporation of covariates. Three regression models were constructed as follows: Model 1: Adjusted only for the HRR subgroup; Model 2: Based on Model 1, further adjusted for basic clinical characteristics including age, gender, insulin use, dietary control status, smoking and drinking habits, comorbidities, and BMI; Model 3: Based on Model 2, additionally adjusted for laboratory indicators including HGB, RDW, WBC, LYM, NEU%, ketone bodies, CHOL, CRP, TG, Lac, HbA1c, ALT, AST, SCR, FPG, and LDH.

The results showed that, with the HRR ≤ 2.54 group as the reference, the analysis of Model 1 indicated that the recurrence risks in the HRR ≤ 2.99 group, HRR ≤ 3.44 group, and HRR>3.44 group were all significantly reduced. The adjusted OR values were 0.294 (95% CI: 0.141 - 0.614, P = 0.001), 0.180 (95% CI: 0.085 - 0.382, P < 0.001), and 0.119 (95% CI: 0.053 - 0.266, P < 0.001), respectively.

In Model 2 which controls for the basic clinical characteristics, the negative association between the HRR group and the recurrence risk remained significant, and the effect trend was consistent with that of Model 1: the OR values for the HRR ≤ 2.99 group, HRR ≤ 3.44 group, and HRR > 3.44 group were 0.269 (95% CI: 0.121 - 0.595, P = 0.001), 0.176 (95% CI: 0.079 - 0.394, P < 0.001), and 0.099 (95% CI: 0.040 - 0.244, P < 0.001), respectively.

In Model 3 which further incorporates laboratory indicators, the aforementioned association still holds statistical significance. The OR values for the HRR ≤ 2.99 group, HRR ≤ 3.44 group, and HRR > 3.44 group are 0.194 (95% CI: 0.061 - 0.616, P = 0.005), 0.131 (95% CI: 0.036 - 0.476, P = 0.002), and 0.050 (95% CI: 0.012 - 0.212, P < 0.001), respectively.

**Figure 2:**
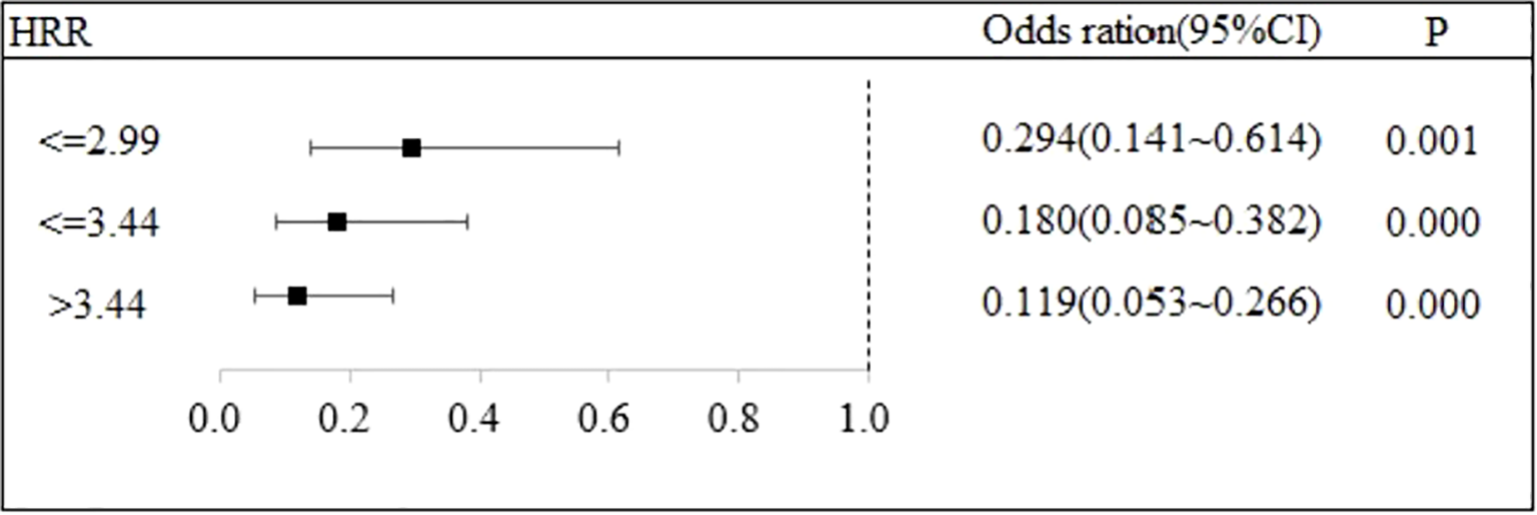
The association between HRR and recurrence of DKA.

**Figure 3:**
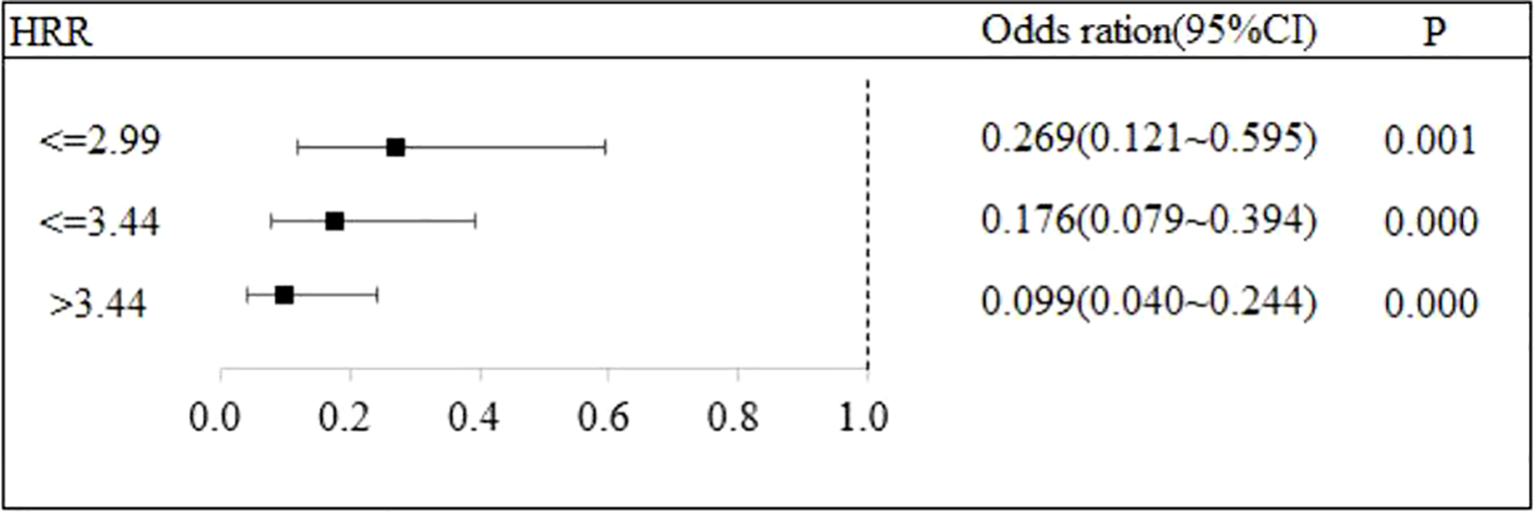
The association between HRR and recurrence of DKA.

**Figure 4:**
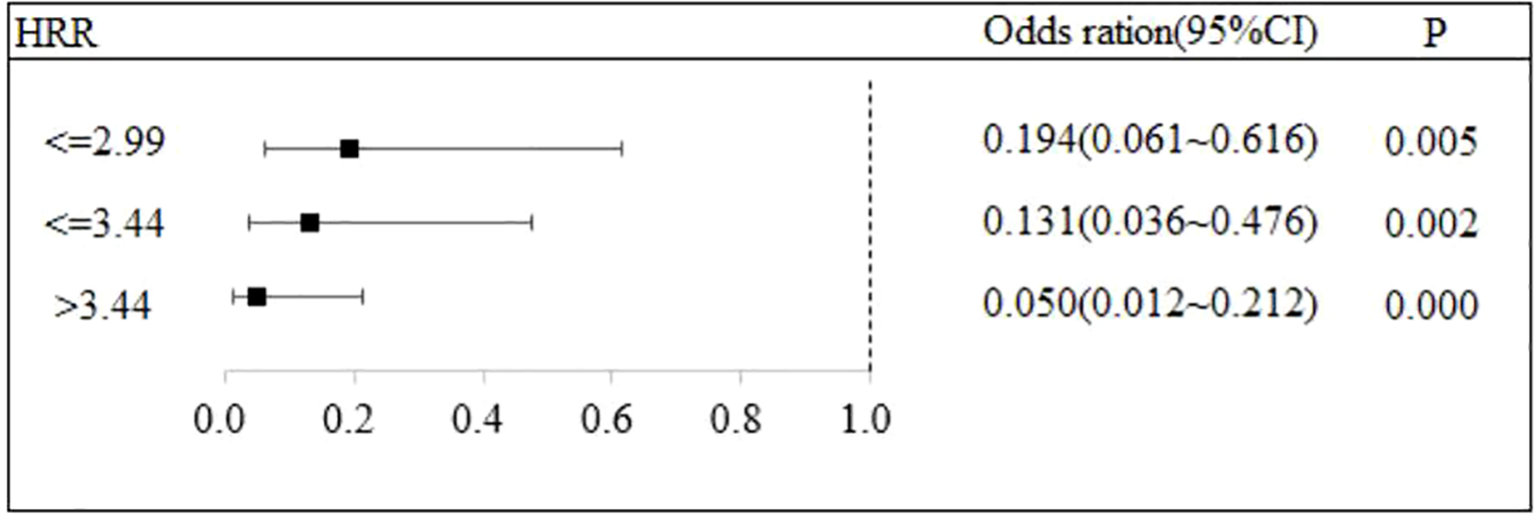
The association between HRR and recurrence of DKA.

An increase in the HRR was significantly associated with a reduced risk of DKA recurrence (P < 0.05). Notably, this association remained statistically stable even after the stepwise adjustment of basic clinical characteristics (Model 2) and additional laboratory indicators (Model 3). These findings indicate that HRR can serve as an independent predictor of DKA recurrence risk, and the results of the regression analysis exhibit good robustness. Detailed regression results are presented in **Table 3**, and supplementary visualizations are provided in **Figures 2**, **3**, **4**.

**Table 3:** The impact of HRR on recurrence.

Model	HRR	OR	95%CI	P
Model 1	<=2.54	1	–	–
	<=2.99	0.294	0.141~0.614	0.001
	<=3.44	0.180	0.085~0.382	0.000
	>3.44	0.119	0.053~0.266	0.000
Model 2	<=2.54	1	–	–
	<=2.99	0.269	0.121~0.595	0.001
	<=3.44	0.176	0.079~0.394	0.000
	>3.44	0.099	0.040~0.244	0.000
Model 3	<=2.54	1	–	–
	<=2.99	0.194	0.061~0.616	0.005
	<=3.44	0.131	0.036~0.476	0.002
	>3.44	0.050	0.012~0.212	0.000

### The value of HRR in predicting the recurrence rate

**Figure 5:**
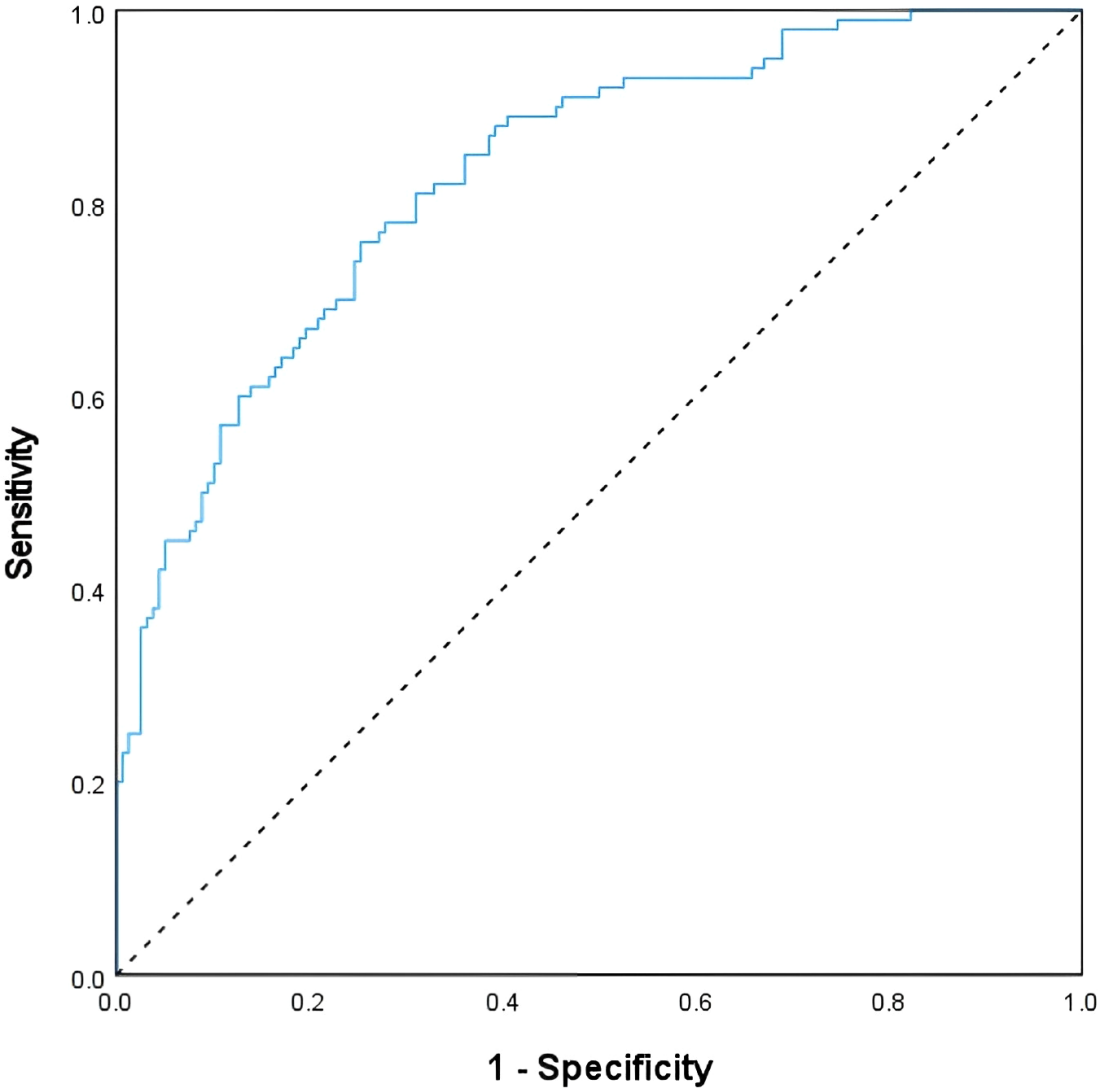
Value of HRR ratio in predicting DKA recurrence.

Concurrently, we evaluated the predictive value of HRR for DKA recurrence using ROC curve analysis. The ROC curve results showed that the optimal cutoff value of HRR for predicting DKA recurrence was 2.99. At this threshold, the sensitivity was 76.00%, the specificity was 74.70%, and the area under the ROC curve (AUC) was 0.830 (95% confidence interval [CI]: 0.780–0.880), indicating a good predictive performance of HRR for DKA recurrence. As shown in **Figure 5**.

## Discussion

In the present study, we explored the predictive value of the combined application of HGB and RDW for DKA recurrence in patients with T2DM. Both HGB and RDW are well-established biomarkers: HGB reflects red blood cell functional integrity, while RDW indicates red blood cell size heterogeneity, and together they have been widely used to evaluate red blood cell damage and predict disease prognosis in various clinical settings. Notably, in recent years, the HRR, has garnered increasing attention as a novel composite biomarker. Its unique advantage lies in its ability to integrate information on red blood cell health, systemic inflammatory status, and nutritional conditions, making it a more comprehensive and clinically relevant indicator compared to HGB or RDW alone (13–15). Therefore, the identification of HRR as a predictive marker holds important implications for the clinical management of T2DM patients with DKA. As a composite indicator, HRR integrates insights into red blood cell function and systemic inflammatory responses, thereby shedding light on the potential pathophysiological mechanisms underlying DM and DKA—particularly their intricate associations with inflammation and anemia. Accumulating evidence supports the involvement of inflammation in both the pathogenesis and progression of DM and DKA: pro-inflammatory cytokines (TNF-α) can impair insulin secretion in diabetic patients, while hyperglycemia, in turn, modulates hemodynamic parameters such as blood viscosity and exacerbates inflammatory cascades, ultimately leading to red blood cell damage. During the onset and progression of DKA, patients typically exhibit an inflammatory response that becomes uncontrolled as the condition deteriorates. Collectively, these observations suggest that persistent or dysregulated inflammatory responses may serve as a key driver of DKA recurrence (16–18). Simultaneously, red blood cell dysfunction can induce oxidative imbalance and propagate inflammatory responses. Conversely, inflammatory mediators can directly damage endothelial cells and red blood cells, triggering vascular sclerosis and multi-organ lesions over time. Thus, the bidirectional interaction between inflammation and red blood cell dysfunction forms a pathological cascade that synergistically promotes the occurrence and progression of DM-related complications, including DKA. Given its ability to integrate information on red blood cell integrity and systemic inflammatory status, HRR is well-positioned to reflect the inflammatory burden and nutritional conditions of patients with DKA. This, in turn, provides a valuable clinical basis for formulating more personalized and targeted treatment strategies to mitigate recurrence risk.

While previous studies have explored the individual roles of RDW and HGB in diabetes mellitus and DKA (10, 19), research focusing on the clinical significance of their ratio (HRR) in this specific patient population remains scarce. Building on this research gap, the present study innovatively applied the HRR to the evaluation of T2DM patients with DKA. Our findings demonstrate that HRR possesses substantial practical value in assessing recurrence risk, thereby providing actionable insights to guide clinical treatment decisions and optimize patient management strategies.

Our research results show that compared with the non-recurrence group, the RDW and HGB of patients in the recurrence group were lower. Meanwhile, CHOL, TG,HbA1c, and LDH level in the recurrence group were significantly higher than those in the non-recurrence group, and the differences were statistically significant (P < 0.05).This indicates that the red blood cell function impairment in the recurrence group is more severe than that in the non-recurrence group, which might be related to the disorder of the blood environment, high extracellular osmolarity, oxidative stress, inflammation and long-term high blood sugar. These factors will affect red blood cells, shortening their lifespan, accelerating the aging process, and causing functional disorders. Long-term high blood sugar levels in the body cause the formation of HbA1c molecules within red blood cells. This process results in the production of HbA1c, whose level reflects the average blood sugar level over a longer period of time. An elevated HbA1c level indicates poor glycemic control in patients with a recurrence of DKA. At the same time, the changes in red blood cell properties not only affect their primary oxygen transport function, but also play a significant role in the development and progression of diabetic complications, especially those related to microvascular dysfunction (20, 21). The inflammatory response in DKA is not a simple systemic inflammatory activation, but rather a complex process intertwined with multiple factors, including hyperglycemia, ketoacidosis, oxidative stress, endothelial dysfunction, and abnormal red blood cell metabolism. This inflammatory response is mainly characterized by “low-grade chronic inflammation” — a state that may not induce a significant elevation of conventional inflammatory markers, yet is sufficient to affect the production, metabolism, and function of red blood cells, thereby leading to changes in HRR levels. Therefore, the conventional inflammatory markers (CRP and WBC) in our study mainly reflect short-term acute inflammatory responses and thus fail to capture the differences in this low-grade chronic inflammation. Consequently, no statistically significant differences were observed between the two groups. Furthermore, our findings demonstrate a significant inverse correlation between HRR levels and DKA recurrence rate: as HRR increases, the risk of DKA recurrence decreases progressively. This trend supports the notion that higher HRR values are indicative of better red blood cell functional integrity and a milder systemic inflammatory response, which in turn confer a lower recurrence risk in T2DM patients with DKA. Notably, this result aligns with observations from HRR-related studies in other disease entities. For instance, in patients with peripheral artery disease (PAD), a similar inverse association has been reported: elevated HRR levels are associated with a reduced incidence of PAD, underscoring the potential universality of HRR as a marker reflecting tissue perfusion, inflammatory burden, and cellular functional status across different pathological conditions (8). Furthermore, our ROC curve analysis identified an optimal threshold of 2.99 for HRR in predicting DKA recurrence, with a sensitivity of 76.00%, specificity of 74.70%, and an area under the curve (AUC) of 0.830. Clinicians should pay particular attention to patients with an HRR value < 2.99, as this subset is at a higher risk of DKA recurrence and requires timely implementation of targeted preventive measures. Specifically, clinical management should focus on mitigating key precipitating factors of DKA recurrence, including preventing infections, avoiding interruptions in insulin or hypoglycemic medication therapy, managing stress states, and addressing eating disorders or gastrointestinal disturbances—all of which can exacerbate metabolic derangements and trigger recurrent DKA episodes. For these high-risk patients, more standardized pharmacotherapeutic regimens, individualized diet and exercise plans, adequate hydration to prevent dehydration, and regular blood glucose monitoring are essential to optimize glycemic control and reduce the risk of long-term complications. In summary, HRR serves as a valuable tool for predicting DKA recurrence, with the distinct advantage of being readily accessible in routine clinical practice. Given its simplicity, cost-effectiveness, and robust predictive ability, HRR holds significant promise for clinical application in the risk stratification and personalized management of T2DM patients with DKA.

Our research results have certain limitations. Firstly, this study employed a retrospective design, which cannot fully eliminate selection bias. Specifically, some indicators—including blood gas parameters, thyroid function, hematological diseases, Insulin treatment compliance, glucose level at admission and surgical history—could not be acquired. Additionally, other influencing factors were not taken into account, such as treatment adherence, treatment discontinuation or intolerance, socioeconomic status, acute triggering events (e.g., infections), and the use of SGLT2 inhibitors. These omissions may have led to temporary deviations in the validity of our research findings. Secondly, our focus was solely on the HRR values of patients at the time of their first admission, without tracking the dynamic changes in HRR after admission. Thirdly, we only investigated the predictive value of HRR for DKA recurrence in patients with T2DM, and did not conduct relevant studies on type 1 diabetes mellitus and other types of diabetes. Fourthly, this was a single-center study, with research subjects recruited only from a local medical center. Despite these limitations, our findings still demonstrate that the HRR possesses significant clinical value in predicting DKA recurrence in T2DM patients.

In view of the limitations of this study, the following suggestions for future research are proposed. Firstly, it is recommended to conduct multi-center, large-scale prospective studies that include more relevant indicators, and expand the geographical scope and coverage of research subjects. This approach will help reduce selection bias. Secondly, it is necessary to strengthen the monitoring of dynamic changes in HRR and continuously track patients’ HRR levels at different time points after admission (e.g., one week, one month, and three months after treatment), so as to more comprehensively clarify the predictive value of HRR. Thirdly, it is suggested to expand the research scope by including patients with type 1 diabetes as well as those with specific types of diabetes complicated with DKA. This will help explore the applicability of HRR in predicting DKA recurrence among patients with different types of diabetes. Fourthly, future research can further delve into the potential molecular mechanisms by which HRR affects DKA recurrence in T2DM patients. By combining biological markers such as inflammatory factors and oxidative stress indicators, the pathophysiological association between HRR and DKA recurrence can be elucidated, providing a theoretical basis for clinical intervention.

## Conclusion

In conclusion, the current study demonstrates a significant association between the HRR and DKA recurrence in patients with T2DM. Specifically, a decrease in HRR (< 2.99) is associated with an increased risk of future DKA recurrence.

## Data Availability

The datasets generated and analyzed during the current study are not publicly available due the data pertains to personal privacy, but are available from the corresponding author on reasonable request. Requests to access the datasets should be directed to aqslyyjzk@163.com.
